# A New Family of Multilevel Grid Connected Inverters Based on Packed U Cell Topology

**DOI:** 10.1038/s41598-017-12806-5

**Published:** 2017-09-29

**Authors:** Majid Pakdel, Saeid Jalilzadeh

**Affiliations:** University of Zanjan, Department of Electrical Engineering, Zanjan, Iran

## Abstract

In this paper a novel packed U cell (PUC) based multilevel grid connected inverter is proposed. Unlike the U cell arrangement which consists of two power switches and one capacitor, in the proposed converter topology a lower DC power supply from renewable energy resources such as photovoltaic arrays (PV) is used as a base power source. The proposed topology offers higher efficiency and lower cost using a small number of power switches and a lower DC power source which is supplied from renewable energy resources. Other capacitor voltages are extracted from the base lower DC power source using isolated DC-DC power converters. The operation principle of proposed transformerless multilevel grid connected inverter is analyzed theoretically. Operation of the proposed multilevel grid connected inverter is verified through simulation studies. An experimental prototype using STM32F407 discovery controller board is performed to verify the simulation results.

## Introduction

Multilevel inverters are the most suitable solution for grid connection of renewable energy resources such as photovoltaic arrays (PV) or wind turbines. Multilevel inverters can produce high voltage with lower harmonics and lower stress on power switches^1^. The most common multilevel inverter topologies are cascaded H-bridge (CHB), neutral point clamped (NPC), and flying capacitors (FC)^2–4^. Packed U cells (PUC) are recent developed multilevel inverters with reduced number of capacitors and power devices^5–10^. The PUC topology needs only one high voltage DC power supply and the lower voltage levels are automatically generated with switching operation in packed U cell capacitors. However, the CHB inverter requires many DC power sources^11^. With equal number of voltage levels, the PUC inverter requires half of power supplies and one third of capacitors compared with FC topology^12^. Furthermore, in the PUC inverter the zero voltage level in freewheeling period can be achieved without using extra power switches. Therefore, the common mode voltage (CMV) will be zero in freewheeling period; as a result the ground leakage current will be declined significantly.

In this paper a novel PUC based multilevel inverter is proposed. Unlike the PUC topology, a lower DC power supply from renewable energy resources such as photovoltaic arrays (PV) is used as a base power source. In fact, other power sources are extracted from the base lower DC power supply using isolated DC-DC power converters. In this case, the output peak voltage will be the summation of lower DC power supply and individual DC power sources extracted from the base power source via isolated DC-DC power converters. Moreover, the DC source voltages are the integer multiples of the lowest DC power supply. Therefore, in contrast with the PUC topology with a higher DC voltage source, in the proposed multilevel inverter, multiple low voltage DC power supplies are used.

Moreover, only a lower DC source and multiple isolated push-pull or forward DC-DC converters can be used instead of a higher DC voltage supply in the PUC topology. In this paper a novel packed U cell (PUC) based multilevel grid connected inverter is elaborated on. The U cell arrangement consists of two power switches and one capacitor. However, in the proposed converter topology, a lower DC power supply from renewable energy resources such as photovoltaic arrays (PV) is used and the other voltages across capacitors are obtained from isolated DC-DC power converters. The proposed topology offers higher efficiency and lower cost applying a small number of power switches and only one lower DC power source. Other capacitor voltages are obtained from the base lower DC power source using isolated DC-DC power converters. After analyzing the operation principle of proposed transformerless multilevel grid connected inverter theoretically, operation of the proposed multilevel grid connected inverter is verified through simulation studies. To validate the simulation results, an experimental prototype using STM32F407 discovery controller board is performed.

## Proposed Multilevel Inverter

**Figure1:**
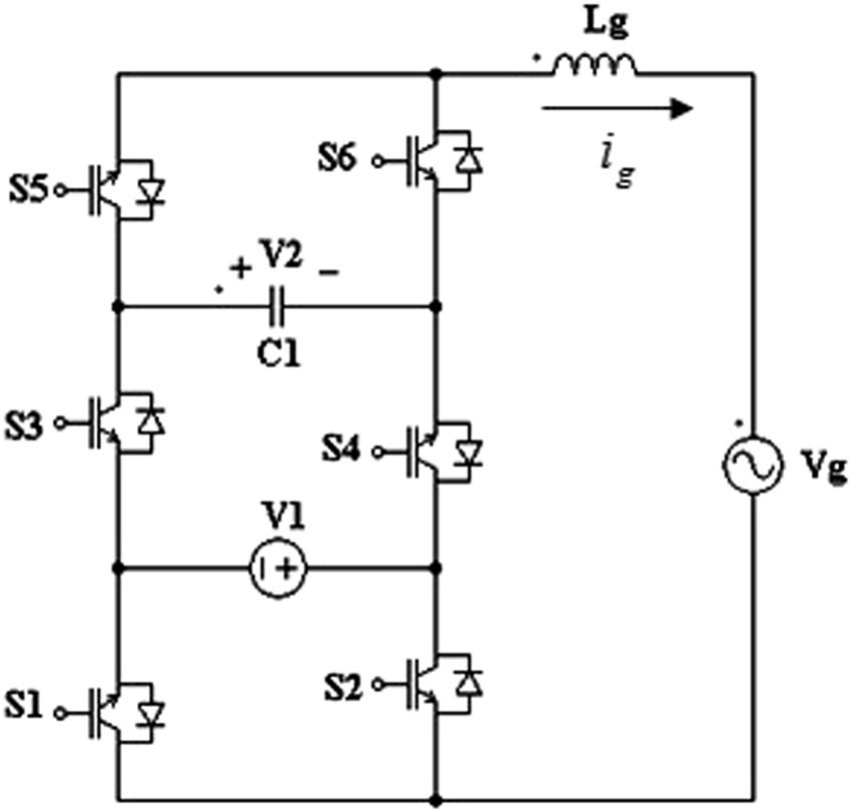
Proposed 3-cell PUC based grid connected inverter.

The proposed single phase three-cell PUC based transformerless grid connected 7-level inverter topology is depicted in Fig. 1. The proposed inverter topology consists of three cells separated by one DC power source and a capacitor. Voltage V_1_ is the DC power supply generated from renewable resources such as photovoltaic (PV) panels and the voltage V_2_ is a voltage produced by an isolated push pull or a forward DC-DC converter which is connected to the low voltage V_1_ of PV panels. In the proposed 7-level inverter topology, the following equation is satisfied.

1 $${{\boldsymbol{V}}}_{2}=2{{\boldsymbol{V}}}_{1}$$

The switching function of switch S_i_ is defined as follows.

2 $${s}_{i=1,2,\mathrm{...},6}=\{\begin{array}{c}1,\,if\,{S}_{i}\,is\,ON\\ 0,\,if\,{S}_{i}\,is\,OFF\end{array}$$

Depending on the control states s_i_, the output voltage level is determined. Eight possible switching states are identified as illustrated in Table 1. As it can be viewed from Table 1, the states 4 and 5 are representing the zero voltage vectors; however, the other six states are active vectors generating appropriate voltage levels at the output of grid connected multilevel inverter.

**Table 1:** Switching table of proposed 3-cell PUC based grid connected 7-level inverter.

State	V_AN_	s_1_	s_2_	s_3_	s_4_	s_5_	s_6_
1	V_1_ + V_2_	1	0	0	1	1	0
2	V_2_	0	0	0	1	1	0
3	V_1_	1	0	0	1	0	0
4	0	0	0	0	1	0	0
5	0	0	0	1	0	0	0
6	−V_1_	0	1	1	0	0	0
7	−V_2_	0	0	1	0	0	1
8	−V_1_ − V_2_	0	1	1	0	0	1

Using Kirchhoff laws the following equation between the voltages V_1_ and V_2_, grid current i_g_, and the switching states s_i_ could be written.

3 $$({s}_{5}-{s}_{6}){V}_{2}+({s}_{1}-{s}_{2})\,{V}_{1}={L}_{g}\frac{d{i}_{g}(t)}{dt}+{V}_{g}(t)$$

**Figure 2:**
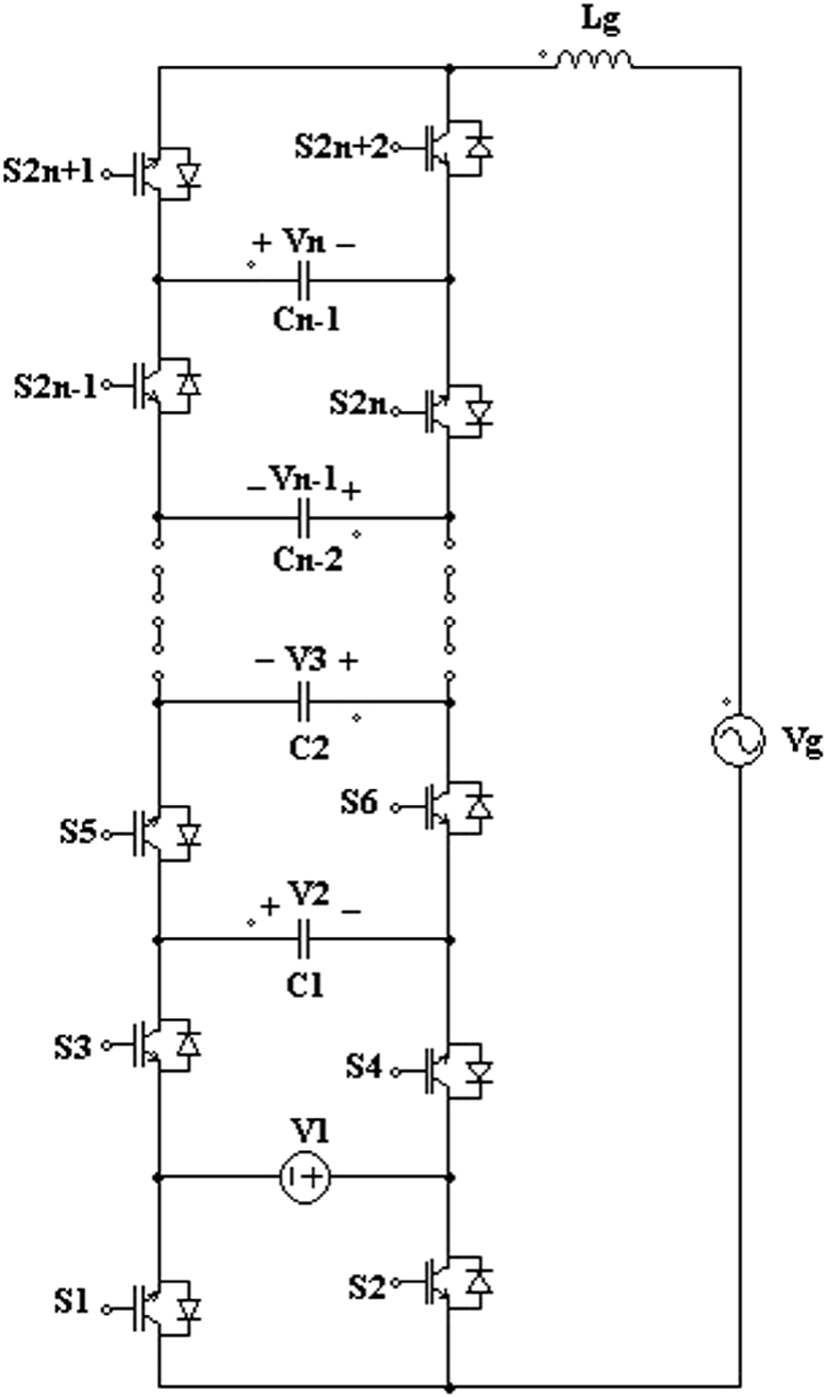
Proposed (n + 1)-cell PUC based n(n + 1) + 1-level inverter.

For the proposed 3-cell PUC based grid connected 7-level inverter, the grid current must be controlled for ensuring proper operation of the 3-cell PUC based multilevel inverter. Generally, the proposed 3-cell PUC based 7-level inverter can be extended to more levels and lower DC power sources by adding more cells to the PUC based multilevel inverter. Figure 2 illustrates an n-DC power supply and (n + 1) cells based on the proposed multilevel inverter. In this case, the total voltage levels (TVL) are calculated as follows.

4 $${\boldsymbol{TVL}}={\boldsymbol{n}}({\boldsymbol{n}}+1)+1$$

where, n is the total number of DC power sources in the proposed PUC based multilevel inverter. Moreover, the voltage relation between n-DC power sources is expressed with the following equation.

5 $$\{\begin{array}{c}{V}_{1}=\,V\\ {V}_{2}=\,2\,V\\ {V}_{3}=\,3\,V\\ \,\mathrm{.......}\\ {V}_{n-1}=\,(n-1)\,V\\ {V}_{n}=\,n\,V\end{array}$$

Therefore, the peak output voltage level is written as the following equation.

6 $${{\boldsymbol{V}}}_{{\boldsymbol{peak}}}=\,{{\boldsymbol{V}}}_{1}+\,{{\boldsymbol{V}}}_{2}\,+\,\mathrm{...}\,+\,{{\boldsymbol{V}}}_{{\boldsymbol{n}}}=\,\frac{{\boldsymbol{n}}\,({\boldsymbol{n}}+1)}{2}{\boldsymbol{V}}$$

## Operation of the Proposed 3-Cell PUC Based 7-Level Grid Connected Inverter

**Figure 3:**
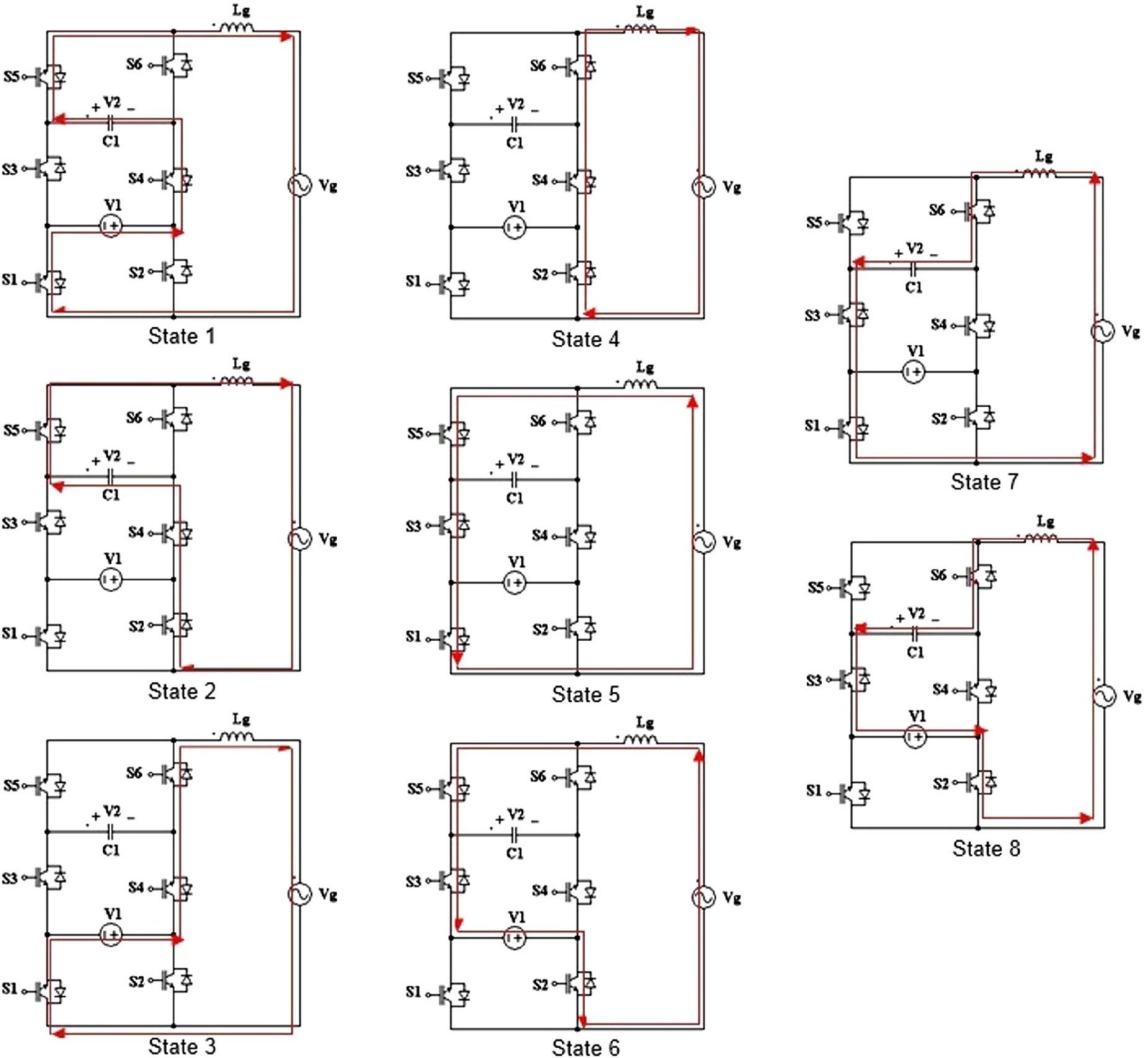
One-cycle operating states of the proposed packed U cell based 7-level grid connected inverter.

One-cycle operating states of the proposed 3-cell PUC based 7-level grid connected inverter are illustrated in Fig. 3. The first DC power source V_1_ which is generated from PV panels, and the second DC power supply V_2_ whose voltage is twice as much as voltage V_1_ and is built with DC source V_1_ using isolated push pull or forward DC-DC converters, are required to produce the designed voltage levels across the grid voltage. Six power devices S_1,2,3,4,5,6_ have been used in the proposed multilevel inverter. Each power switch consists of an IGBT with its anti-parallel diode and has two operating states i.e. ON state and OFF state. As shown in Table 1 and Fig. 3 eight possible operating states can be identified in the proposed 3-cell PUC based 7-level grid connected inverter. Two states 4 and 5 are representing the zero voltage state and the other six states are providing suitable voltage levels across the grid voltage as depicted in Table 1. The operation sequence of the proposed grid connected multilevel inverter is defining eight possible states as portrayed in Fig. 3. As shown in Fig. 3, the grid voltage is fed by seven voltage levels i.e. V_1_ + V_2_, V_2_, V_1_, 0, −V1, −V_2_ and −V_1_ − V_2_ in the single phase topology.

## Control Strategy of the Proposed 3-Cell PUC Based 7-Level Grid Connected Inverter

**Figure 4:**
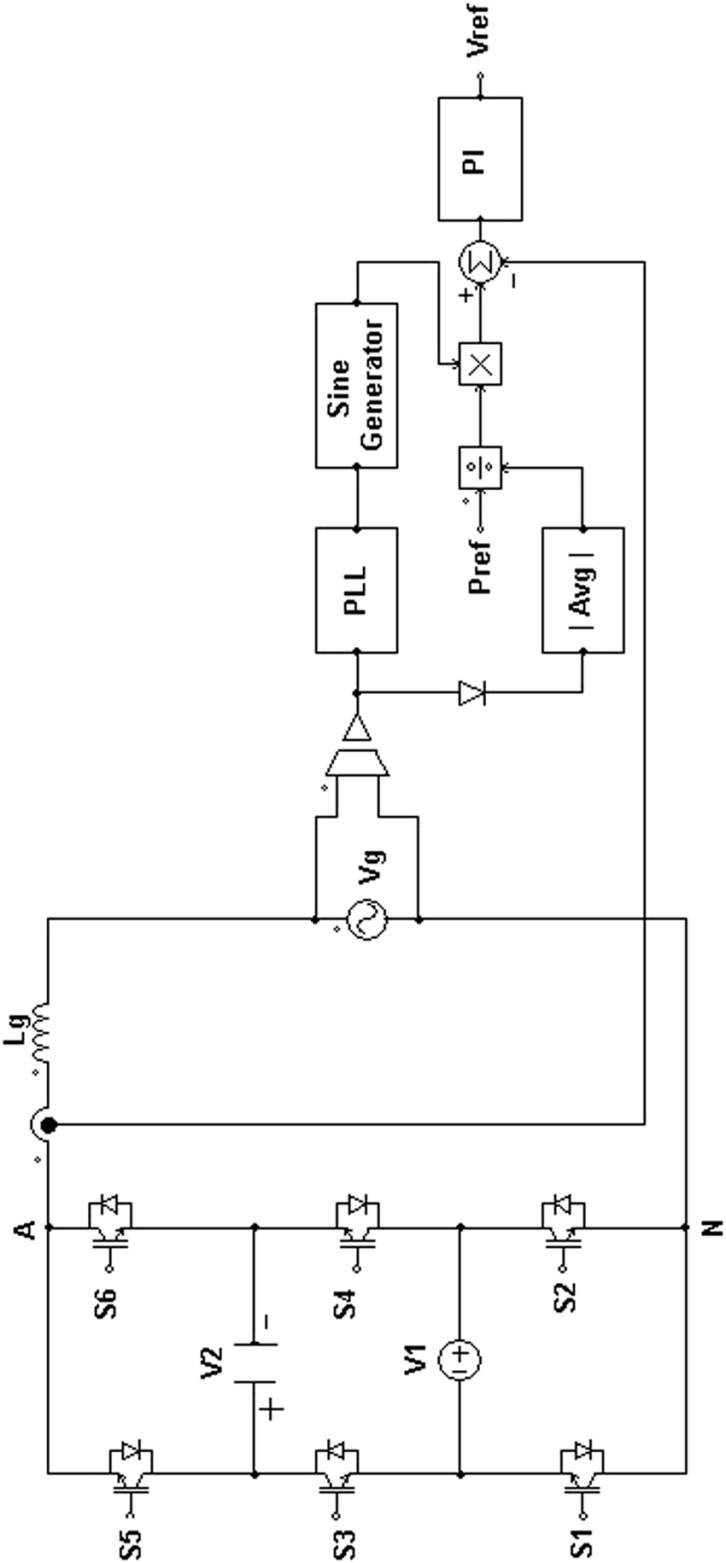
Control strategy for generating the reference voltage for the proposed PUC based multilevel inverter.

The control strategy for generating the reference voltage for the proposed PUC based multilevel inverter is depicted in Fig. 4.

**Figure 5:**
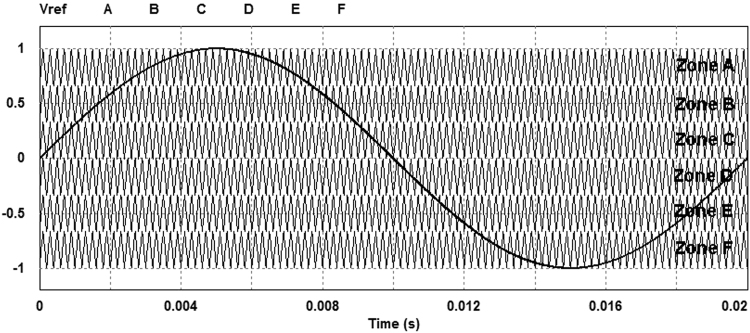
Seven-level sinusoidal modulation.

**Figure 6:**
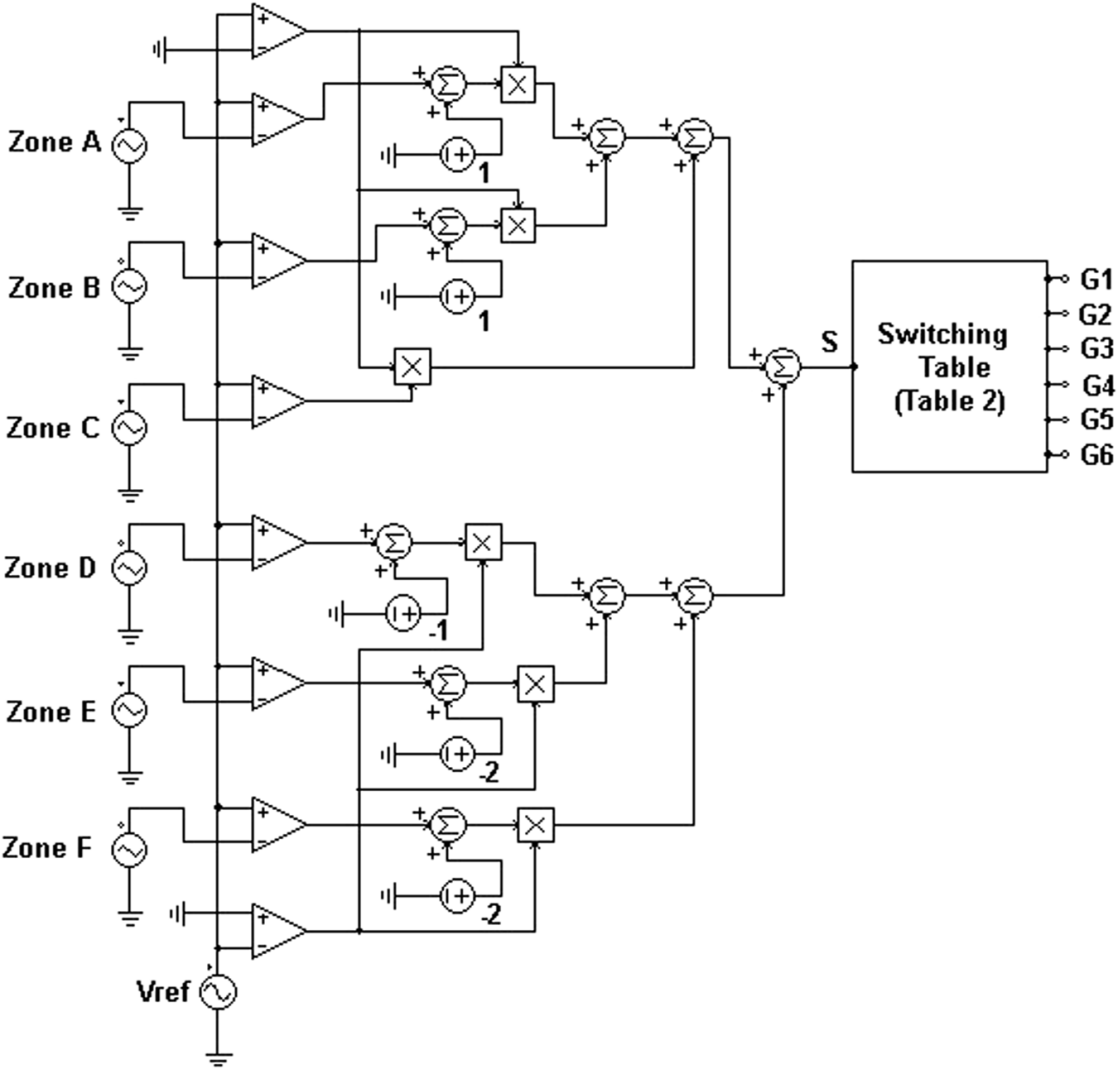
Proposed modulation method.

**Figure 7:**
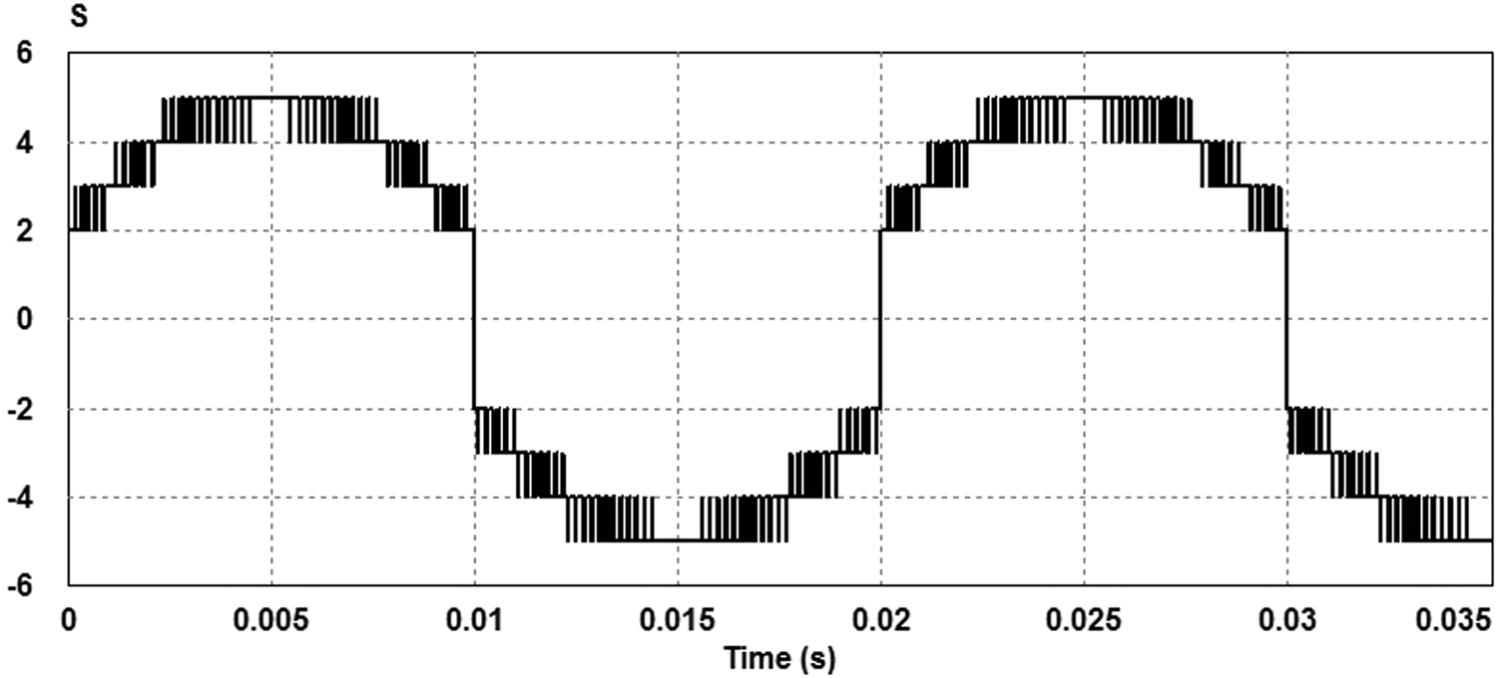
Signal S waveform.

Assuming the reference voltage to be a sinusoidal waveform, it can be subdivided into three positive and three negative zones as illustrated in Fig. 5. Using seven-level sinusoidal modulation, four pulses can be produced in whose positive zones, the comparator output (one or zero) is added to 1 and in negative zones the comparator output (one or zero) is added to −2. However, in Zone D (or state 5) the comparator output (one or zero) is added to −1 as shown in Fig. 6. Summing these signals will produce signal S which has eight voltage levels as illustrated in Fig. 7.

The voltage levels correspond to the seven-level output voltages with the redundant zero output voltages (states 4 and 5). Therefore, using a lookup table (Table 2), the seven voltage levels would be synthesized as shown in Fig. 6.

**Table 2:** Lookup table of proposed 3-cell PUC based grid connected 7-level inverter.

State	Signal S	V_AN_	s_1_	s_2_	s_3_	s_4_	s_5_	s_6_
1	5	V_1_ + V_2_	1	0	0	1	1	0
2	4	V_2_	0	0	0	1	1	0
3	3	V_1_	1	0	0	1	0	0
4	2	0	0	0	0	1	0	0
5	−2	0	0	0	1	0	0	0
6	−3	−V_1_	0	1	1	0	0	0
7	−4	−V_2_	0	0	1	0	0	1
8	−5	−V_1_ − V_2_	0	1	1	0	0	1

## Simulation Results

**Figure 8:**
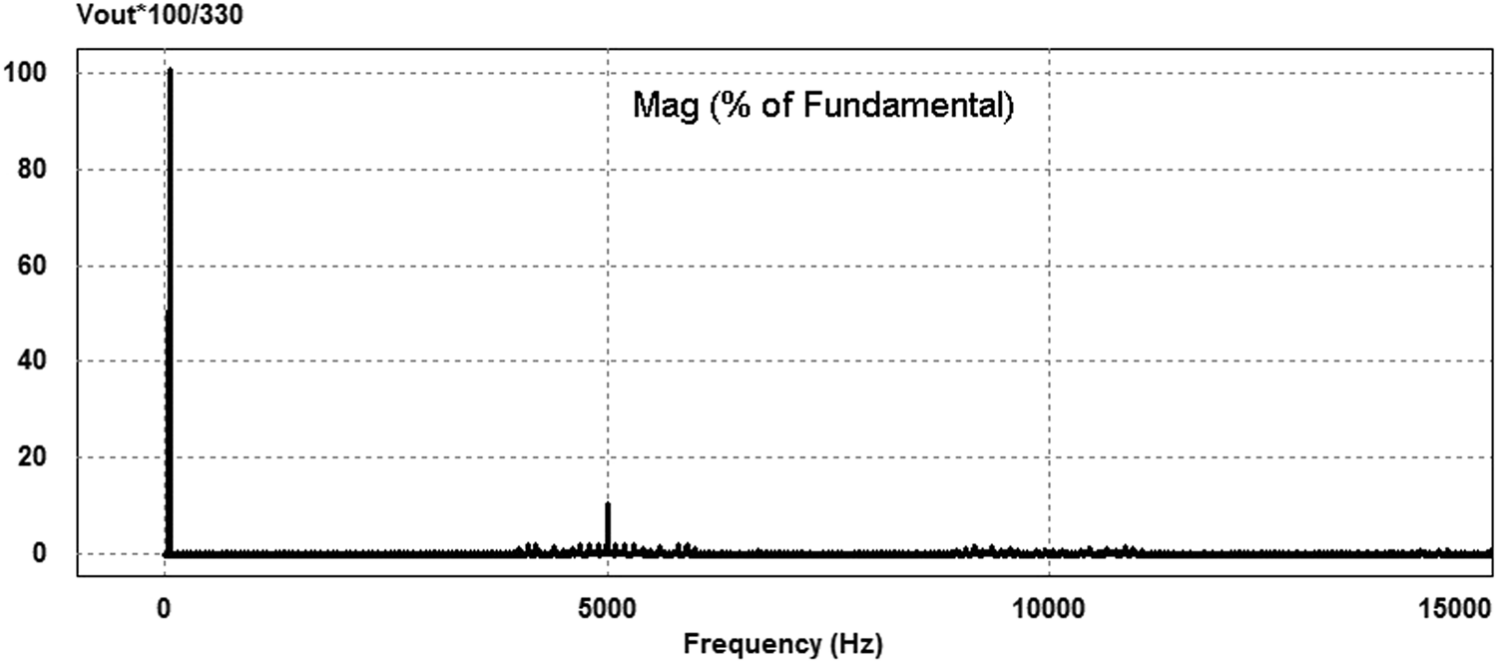
Harmonic contents of the output voltage.

**Figure 9:**
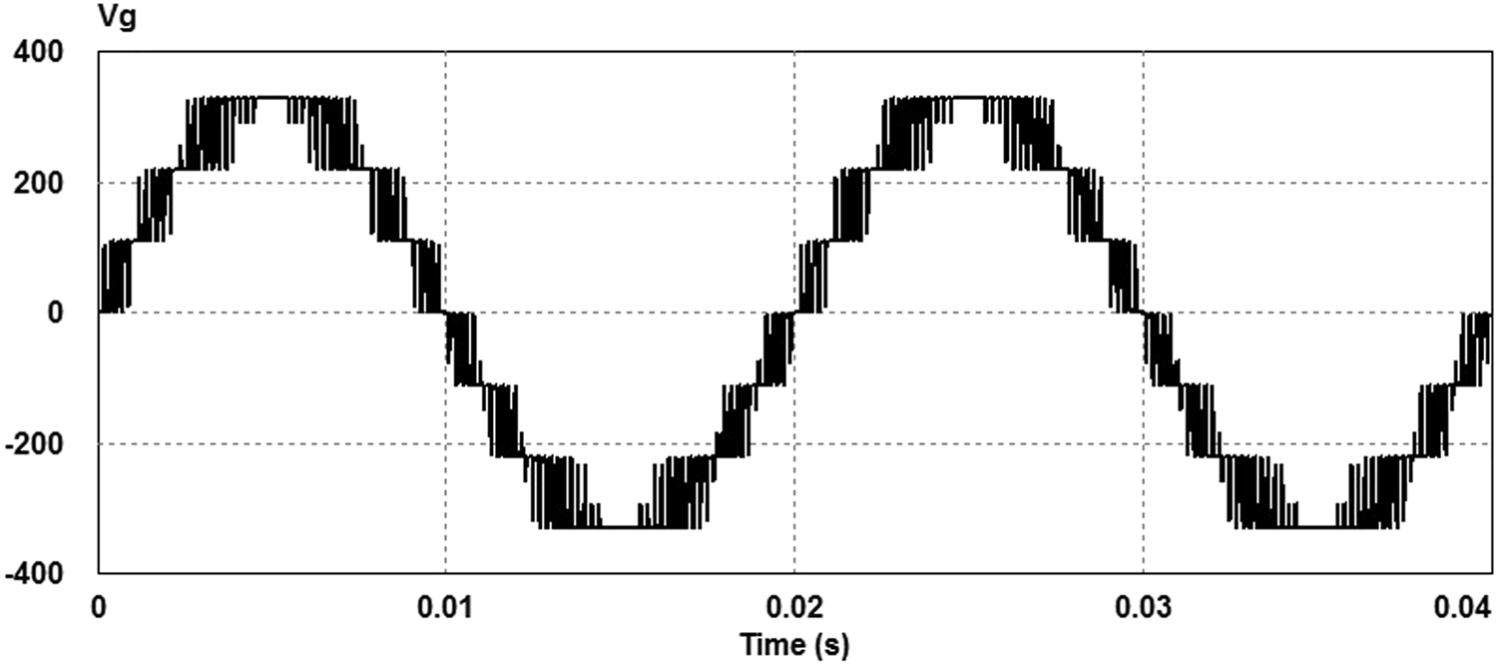
Seven level output voltage.

The proposed 3-cell PUC based grid connected 7-level inverter parameters are given in Table 3. The V_1_ dc bus is assumed to be 110 V and the V_2_ dc bus is generated from an isolated push pull DC-DC converter or a forward converter. The voltage V_2_ is set to be twice as big as the voltage V_1_ value (220 V). The AC load is a series connection of a 100 Ω resistor and an inductor of 1 mH. The switching frequency of the sinusoidal PWM modulation is set at 5 kHz. The simulation was performed using PSIM software. The AC load voltage has seven levels and its harmonic contents are around multiples of the switching frequency (5 kHz) as shown in Fig. 8. The calculated total harmonic distortion (THD) of the output voltage is 14.76%. Furthermore, as illustrated in Fig. 8, the output voltage fundamental component amplitude with the frequency of 50 Hz is equal to 330 V. The seven level output voltage is depicted in Fig. 9.

**Table 3:** Simulation parameters.

Parameters	Values
V_1_ DC bus	110 V
V_2_ DC bus	220 V
Load inductance	1 mH
Load Resistor	100 Ω
AC supply network	~220 V
Modulating frequency	5 kHz

**Figure 10:**
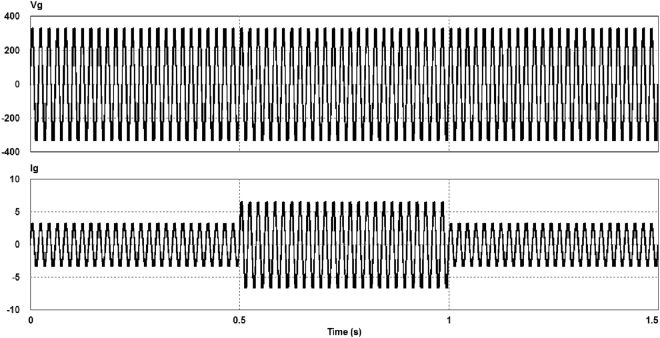
Waveforms of the output voltage v_g_ and load current i_g_ in transient operation.

In order to evaluate the system dynamics, a sudden resistance load change from 100 Ω to 50 Ω is applied at time t = 0.5 s. At time t = 1 s the load resistance is changed from 50 Ω to 100 Ω again. Figure 10 illustrates good dynamic responses during sudden load variations for the output voltage and current.

## Experimental Results

The Matlab Waijung Simulink blockset is used to automatically generate STM32F407 discovery ARM based microcontroller bin code from Simulink block diagrams. The code is used to implement the proposed multilevel grid connected control strategy. An electronic circuit is implemented for sensing analog voltage and current signals.

**Figure 11:**
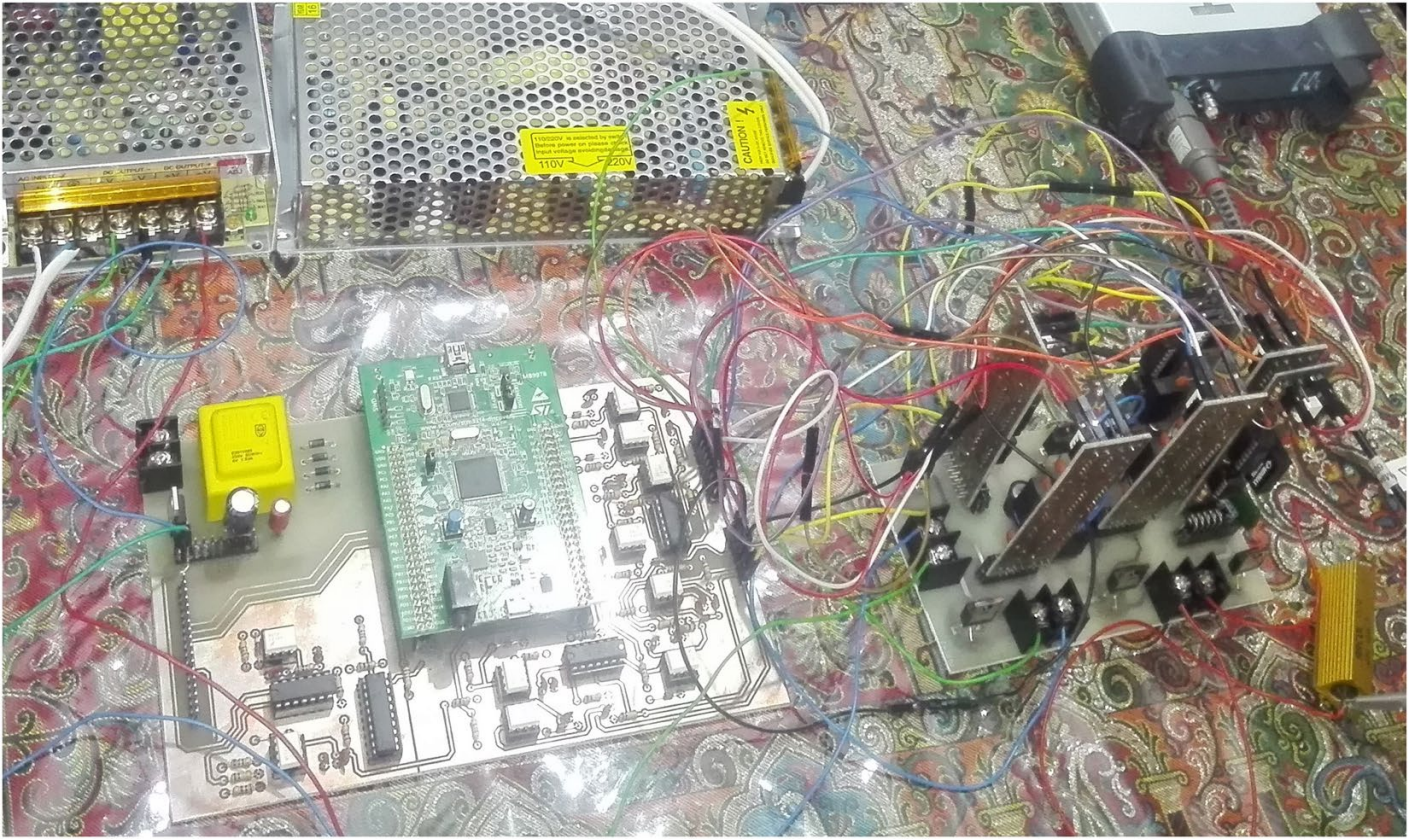
Experimental prototype of the proposed multilevel grid connected inverter.

**Figure 12:**
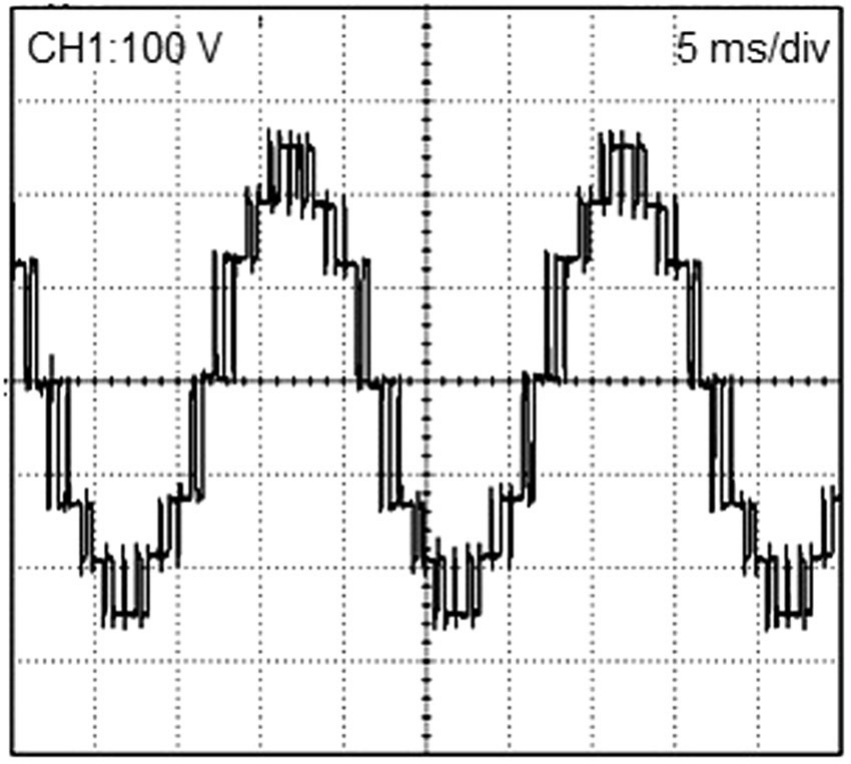
7-level output voltage of the proposed 3-cell PUC based grid connected inverter.

Six digital PWM signals and corresponding gate driver electronic circuits are used to generate the IRF840 MOSFET gate driving pulses. The circuit components values are as selected in the simulation results section. Figure 11 illustrates an experimental prototype of the proposed 3-cell PUC based grid connected 7-level inverter. A high concordance between simulation and experimental results of the proposed multilevel grid connected inverter could be concluded. The seven level output voltage is depicted in Fig. 12, which is similar to the simulation result shown in Fig. 9.

## Conclusion

In this paper a novel packed U cell (PUC) based multilevel grid connected inverter was presented. Unlike the U cell arrangement which consists of two power switches and one capacitor, in the proposed converter topology, a lower DC power supply from renewable energy resources such as photovoltaic arrays (PV) is used as a base power source. The proposed topology offers higher efficiency and lower cost using a small number of power switches and a lower DC power source which is supplied from renewable energy resources. Other capacitor voltages are extracted from the base lower DC power source using isolated DC-DC power converters. The operation principle of proposed transformerless multilevel grid connected inverter was analyzed theoretically. Operation of the proposed multilevel grid connected inverter was verified through simulation studies. An experimental prototype using STM32F407 discovery controller board was performed to validate the simulation results.
